# Editorial: Viral Evasion Mechanisms of the Host Response

**DOI:** 10.3389/fcimb.2020.00090

**Published:** 2020-02-28

**Authors:** Ricardo Martín Gómez, Eugenio Antonio Carrera Silva, Jônatas Santos Abrahão, Siew Pheng Lim, Aleem Siddiqui

**Affiliations:** ^1^Laboratorio de Virus Animales, Instituto de Biotecnología y Biología Molecular, CONICET-Universidad Nacional de La Plata, La Plata, Argentina; ^2^Global Viral Network, Baltimore, MD, United States; ^3^Laboratorio de Trombosis Experimental, Instituto de Medicina Experimental, CONICET-Academia Nacional de Medicina, Buenos Aires, Argentina; ^4^Laboratorio de Virus, ICB, Universidade Federal de Minas Gerais (UFMG), Belo Horizonte, Brazil; ^5^Denka Life Innovation Research, Singapore, Singapore; ^6^Division of Infectious Diseases, School of Medicine, University of California, San Diego, La Jolla, CA, United States

**Keywords:** DNA virus, RNA virus, MicroRNA, programmed cell death protein, microbiota, RNAstasis, cell metabolism

An essential function of the host response is to protect the organism against invading pathogens. At present, a multiplicity of mechanisms has been described on how the host sense and response to virus infections. Viruses are intracellular pathogens. Both RNA and DNA viruses have evolved mechanisms to evade host detection and to blunt both the host innate and adaptive immune responses. Considering viruses as pathogens with a relatively fast evolutionary rate, particularly RNA viruses, the result of host-virus coevolution depends on the rapid recognition and response by the host as well as on the evasion mechanism by the virus as a continuous struggle for escape/spread and immunity/clearance of virus from the host.

In this Special Research Topics issue on the recent advances in Viral Evasion Mechanisms of the Host Response, we compiled a total of twelve research and review articles. The special issue includes five Original Research Articles, five Review Articles, and two Mini Review Articles. Meanwhile five articles were dedicated to viral general mechanisms, seven were specifically focused on picornavirus, respiratory syncytial virus (RSV), Dengue virus (DENV), herpes simplex virus (HSV), and Influenza virus (IAV). The family *Picornaviridae* includes some of the most important RNA viruses for human and veterinary diseases as poliovirus, rhinovirus, and foot-and-mouth-disease virus, which comprised pioneer studies on the structural aspects of viral components. In their minireview, Cifuente and Moratorio summarize genetic variation mechanisms used by picornaviruses on structural changes involved in binding receptor and capsid antibody evasion of enteroviruses to ensure adaptation, spread and survival. The *Paramyxoviridae* family includes several important human RNA virus as Measles, Mumps, and RSV. In their article, Bakre et al. hypothesize that viral quasi-species enable RNA viruses to modulate host gene expression by regulating miRNA function via sequence complementarity or identity with the miRNA seed sites and consequently they test the hypothesis by analyzing Paramyxovirus transcripts that mimic or bind to host miRNAs by seed sequence and found that complex molecular interactions likely occurred at this host-virus interface. The human RSV (hRSV) belongs to the *Pneumoviridae* subfamily and is the leading cause of severe acute lower respiratory tract infections in humans at all ages and is the main cause of hospitalization due to pneumonia, asthma, and bronchiolitis in infants. Vaccine development against this pathogen has been delayed after the detrimental effects observed in children by vaccination with a formalin-inactivated hRSV preparation, which caused enhanced disease upon natural viral infection. In this issue, Acevedo et al., discuss the eventual role of Fcγ receptor-mediated immunity underlying such disease exacerbation and immune response mechanisms involved in reinfections. Respiratory viral infections are associated to IgA nephropathy (IgAN). Here, Hu et al., using a RSV-induced IgAN exacerbation mouse model demonstrates that RSV activates C5a-C5aR1 axis and modulates the Th1, Th17, and Treg balance. C5aR1 inhibition alters both kidney damage and Th1, Th17, and Treg cell dysfunction supporting that blocking the C5a-C5aR1 axis might be a potential therapy for RSV-induced IgAN. The Dengue virus (DENV), a member of the *Flaviviridae* family, causes 400 million infections each year. Whereas, primary dengue infection by any of the four serotypes is asymptomatic or mild, secondary infection with a heterotypic serotype is associated with hemorrhagic fever suggesting that pre-existing immunity to DENV play a role for enhanced secondary infections. Ripoll et al. carried out molecular simulations guided by previous *in vitro* experiments and structural studies to explore the role of antibody fine-specificity, viral conformation, and maturation state on antibody dependent enhancement in the context of primary and secondary DENV infections. The Influenza viruses belong to the *Orthomyxoviridae* family and are major pathogens that affect both humans and animals causing severe respiratory illness, including pneumonia. Neutrophils and macrophages play essential roles in the clearance of influenza virus from lungs, before the onset of virus specific immunity but their uncontrolled recruitment and activation contribute to acute lung injury. In this special issue, Rudd et al. study chemokine receptors expression in a murine model of influenza-induced pneumonia and reported a new set of chemokine receptors that modulates several biological functions of neutrophils. On the other hand, Tao et al. performed a comparative analysis of whole-transcriptome RNA expression between two influenza viruses and discuss how differently expressed genes may be involved in host response and evasion mechanisms. The *Herpesviridae* family includes very important human pathogens as the herpes simplex virus (HSV), associated with mucosal lesions and encephalitis. Tognarelli et al. review and update several mechanisms used by HSV that have been described to evade the host antiviral response. The epithelial surfaces of the human body contain complex communities of microorganisms collectively referred to as microbiota. Since the discovery that gut microbiome instruct host immunity, great attention has been directed to this interaction. In their minireview, Domínguez-Díaz et al., provides a general overview of the pro- and antiviral effects of the microbiota to prevent viruses entry into host cells or to help them to evade the host antiviral immunity. Regulation of RNA homeostasis is a central step in eukaryotic gene expression. From transcription to decay, cellular messenger RNAs (mRNAs) associate with specific proteins in order to regulate their entire cycle, including mRNA localization, translation and degradation. The best characterized of such RNA-protein complexes are Stress Granules (SGs) and Processing Bodies (PBs), which are involved in RNA storage and RNA decay/storage, respectively, and are generally associated with repression of gene expression. Gaete-Argel et al. performed an exhaustive update about how viruses have evolved different mechanisms to counteract SGs and PBs assembly or to use them to his own benefit. Moreno-Altamirano et al. explore how viruses mimic, exploit, and/or interfere with host cell metabolic pathways and how, in doing so, they may evade immune responses. Programmed cell death protein (PD-1) and its ligands have received immense attention because of their role in the evasion of tumor cells from antitumor immunity. However, it has been less appreciated that the PD-1/PD-L1 axis also regulates antiviral immune responses and is therefore modulated by a number of viruses. Here, Schönrich and Raftery update and discuss the current literature regarding this important expanding field.

## Author Contributions

All authors listed have made a substantial, direct and intellectual contribution to the work, and approved it for publication.

### Conflict of Interest

SL is employed by Denka Life Innovation Research. The remaining authors declare that the research was conducted in the absence of any commercial or financial relationships that could be construed as a potential conflict of interest.

